# Supplementary material for: RECoN: Rice Environment Coexpression Network for Systems Level Analysis of Abiotic-Stress Response
Source: Front Plant Sci. 2017 Sep 20;8:1640. doi: 10.3389/fpls.2017.01640 (PMC5611544; doi:10.3389/fpls.2017.01640)
Supplement: Supplementary file 6 [file Presentation_1.pdf]

Supplementary Material

RECoN: Rice Environment Coexpression Network for Systems Level Analysis of Abiotic-Stress Response

Arjun Krishnan[1,3,4], Chirag Gupta[2], Madana M.R. Ambavaram[1,5], Andy Pereira[1,2,*].

[1]Virginia Bioinformatics Institute, Virginia Tech, Blacksburg, VA 24061.

[2]Crop, Soil, and Environmental Sciences, University of Arkansas, Fayetteville, Arkansas 72701.

Present Address:

[3]Computational Mathematics, Science, and Engineering, Michigan State University, MI 48824

[4]Biochemistry and Molecular Biology, Michigan State University, MI 48824

[5]Yield10 Bioscience, Inc., 19 Presidential Way, Woburn, MA 01801

[*]**Correspondence:**

Andy Pereira: apereira@uark.edu; phone 4795758435

Plant Sciences Building 115; Crop, Soil, and Environmental Sciences,

University of Arkansas, Fayetteville, Arkansas 72701.

List of Supplementary Material

Supplementary Note

Supplementary Figure 1

Supplementary Figure 2

Supplementary Figure 3

Supplementary Figure 4

Supplementary Figure 5

Supplementary Figure 6

Supplementary Figure 7

Supplementary Figure 8

Supplementary Note

Phenotypic and Physiological Responses to Drought Stress

For drought stress, a set of plants in each of their selected developmental stages (seedlings, vegetative and reproductive) were subjected to progressive drought stress by withholding water, and the degree of drought stress was determined by monitoring the soil moisture content, chlorophyll fluorescence (Fv/Fm and PSII) and the relative water content (RWC) (Supplemental Table S1). The effect of drought stress on photosynthesis was assessed by determination of the chlorophyll fluorescence and the quantum yield of PS II photochemistry (PS II yield) (Table S1). RWC % was used to indicate an integrated plant water status at the given soil moisture level, which also represents water potential, turgor potential, and osmotic adjustment. PS II yield is a measure of the photosynthetic performance of the plant under ambient light conditions, while Fv/Fm is a measure of photo-oxidative to PS II. In this experiment the soil moisture during the dry down was 9.65 % of the controls. At this level of moisture, the RWC %, Fv/Fm, PS II Yield decreased by 67.66 %, 89.04 %, and 62.30 % respectively. All these parameters were significantly different at $LSD_{(0.05)}$.

Supplementary Fig. S1

Gene expression profiles of the 28,421 genes across the 45 conditions/groups, organized based on coexpression cluster membership of genes. For ease of visualization, the genes have been split into 4 groups (blocks) of ~7100 genes each. Conditions indicated on top were clustered based on similarity in expression of all the genes (Pearson correlation; Average linkage) and correspond to the columns in each block. Colors indicate the level of expression (in *log₂* scale) with red, black and green corresponding to a high, moderate and low expression levels

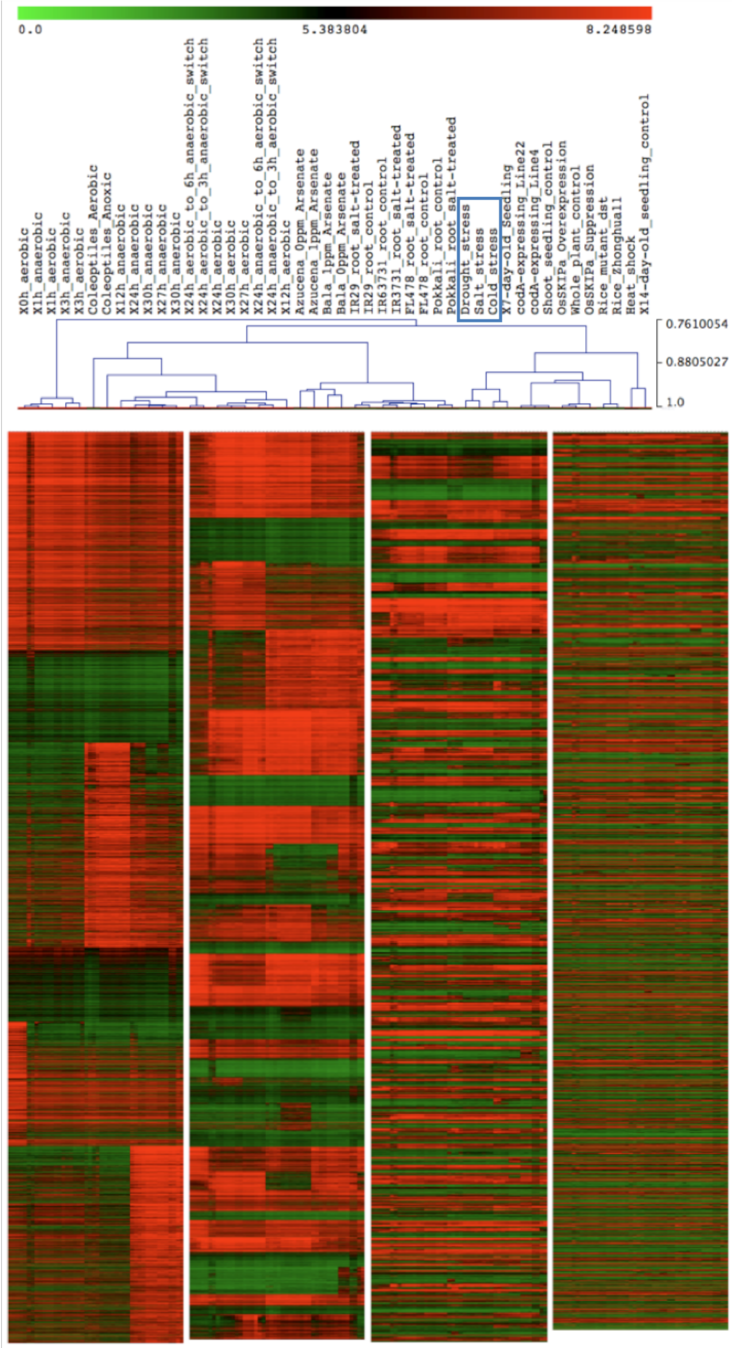

Supplemental Fig. S2

Screenshot of the cluster enrichment results. Clusters significantly enriched in the uploaded transcriptome are listed and linked to their attribute tables. The second column of the table is colored green and orange for positive and negative enrichment, respectively. The last column represent a representative BP term enriched within the cluster (term with the lowest *q*value).

Clusters enriched in uploaded sample

82 clusters found

[click on the cluster ID to see the full list of genes, biological processes, CRE and cytoscape powered graph]

Download this table

▲ ▼	Cluster ▲ ▼	Size ▲ ▼	GSE14275 ▲ ▼	Bio Proc ▲ ▼
1	Cluster0223	25	22.201	response to heat
2	Cluster0520	19	20.533	-
3	Cluster0006	1545	14.948	photosynthesis
4	Cluster0065	108	10.265	translational elongation
5	Cluster0475	17	9.368	-
6	Cluster0005	972	9.221	response to water stimulus
7	Cluster0001	1713	8.467	RNA processing
8	Cluster0079	71	7.727	-
9	Cluster0013	294	6.667	protein import
10	Cluster0328	29	6.630	-
11	Cluster0046	179	6.528	tRNA metabolic process
12	Cluster0072	38	5.893	cellular protein localization
13	Cluster0696	11	5.731	-
14	Cluster0204	20	5.650	cofactor metabolic process
15	Cluster0487	10	5.300	-
16	Cluster0316	23	5.097	cellular component biogenesis
17	Cluster0110	51	4.957	-
18	Cluster0056	138	4.622	cell wall organization

Supplemental Fig. S3

Screenshot of the cluster table with tabs linked to other functional and regulatory annotations.

	Cluster ID	No. Of Genes	Z Score
1	Cluster0223	25	22.201

View Cluster Graph

Process KEGG PATHWAYS Motifs Genes Graph

Gene	Annotation	RAP ID	Arabidopsis homolog	Ontologies	MF	Sdlng. Drought	Veg. Drought	Rep. Drought	GSE14275
LOC_Os03g60780	armadillo/beta-catenin-like repeat containing protein, expressed	Os03g0822700	AT3G09350	see details	Gene	0.8775	0.0000	0.0000	2.0624
LOC_Os04g45480	heat shock protein STI, putative, expressed	Os04g0538000	AT4G12400	see details	Gene	1.1681	0.0000	0.0000	2.0160
LOC_Os06g11610	heat shock 22 kDa protein, mitochondrial precursor, putative, expressed	Os06g0219500	AT5G51440	see details	Gene	1.9542	0.0000	0.0000	3.8412
LOC_Os01g55270	SGS domain containing protein, expressed	Os01g0757500	AT1G30070	see details	Gene	1.4823	0.0000	0.0000	2.7391
LOC_Os01g62290	DnaK family protein, putative, expressed	Os01g0840100	AT3G12580	see details	Gene	1.2597	0.0000	0.6720	3.0820
LOC_Os04g28420	peptidyl-prolyl isomerase, putative, expressed	Os04g0352400	AT3G25230	see details	Gene	1.7990	0.0000	2.8589	8.1557
LOC_Os05g38530	DnaK family protein, putative, expressed	Os05g0460000	AT3G12580	see details	Gene	2.1365	1.6582	0.9521	3.2953
LOC_Os08g40910	expressed protein	Os08g0520600	-	see details	Gene	0.5075	0.0000	0.0000	3.4934
LOC_Os06g46900	phosphosulfolactate synthase-related protein, putative, expressed	Os06g0682900	AT4G21320	see details	Gene	2.9114	3.6168	0.6964	4.1993
LOC_Os04g01740	heat shock protein, putative, expressed	Os04g0107900	AT5G52640	see details	Gene	1.1460	0.0000	1.0353	7.2782
LOC_Os01g04360	hsp20/alpha crystallin family protein, putative, expressed	Os01g0136000	AT1G53540	see details	Gene	2.0138	0.0000	1.1576	4.9196
LOC_Os06g09560	heat shock protein DnaJ, putative, expressed	Os06g0195800	AT3G14200	see details	Gene	0.3110	0.0000	0.0000	4.2053
LOC_Os03g25770	RNA recognition motif containing protein, putative, expressed	Os03g0374575	AT3G13570	see details	Gene	1.0631	1.1425	0.0000	1.2520
LOC_Os06g30970	ubiquitin-conjugating enzyme, putative, expressed	Os06g0506600	AT1G64230	see details	Gene	1.0683	1.2428	0.0000	1.0080
LOC_Os06g12940	expressed protein	Os06g0235900	AT3G28370	see details	Gene	0.0000	0.0000	0.0000	0.7083
LOC_Os09g38100	phosphate carrier protein, mitochondrial precursor, putative, expressed	Os09g0554000	AT2G17270	see details	Gene	0.0000	-0.8564	0.0000	1.4573
LOC_Os02g37000	mitochondrial prohibitin complex protein 1, putative, expressed	Os02g0580500	AT5G40770	see details	Gene	1.8510	3.0020	0.7005	2.8427
LOC_Os10g41930	haloacid dehalogenase-like hydrolase family protein, putative, expressed	Os10g0568900	AT2G38740	see details	Gene	0.0000	0.0000	0.0000	0.5147
LOC_Os01g34480	NAD dependent epimerase/dehydratase family protein, putative, expressed	Os01g0528800	AT5G19440	see details	Gene	0.3049	0.0000	0.0000	0.8718
LOC_Os03g53340	HSF-type DNA-binding domain containing protein, expressed	Os03g0745000	AT2G26150	see details	TF	0.0000	0.0000	0.0000	1.7374
LOC_Os01g18100	OsRhmbd3 - Putative Rhomboid homologue, expressed	Os01g0283500	AT3G17611	see details	Gene	0.0000	0.7715	0.0000	1.0303
LOC_Os03g15960	hsp20/alpha crystallin family protein, putative, expressed	Os03g0266300	AT1G53540	see details	Gene	3.5195	2.5990	1.4322	4.4882
LOC_Os03g16030	hsp20/alpha crystallin family protein, putative, expressed	Os03g0267000	AT1G53540	see details	Gene	2.4040	0.0000	1.5133	6.6780
LOC_Os12g37519	retrotransposon protein, putative, unclassified, expressed	-	-	see details	Gene	0.0000	0.0000	0.0000	2.7478
LOC_Os12g12080	retrotransposon protein, putative, Ty3-gypsy subclass, expressed	-	-	see details	Gene	0.0000	6.2099	0.0000	5.4484

Supplemental Fig. S4

Screenshot of the displayed table for cluster0223 enriched for heat stress response.

	Cluster ID	No. Of Genes	Z Score
1	Cluster0223	25	22.201

View Cluster Graph

Process **KEGG PATHWAYS** **Motifs** **Genes** **Graph**

Cluster	GO ID	# of Genes	GO Description	Score (-log qvalue)
Cluster0223	GO:0009408	21	response to heat	4.186
Cluster0223	GO:0009628	92	response to abiotic stimulus	2.571
Cluster0223	GO:0006457	205	protein folding	1.845

Supplemental Fig. S5

Screenshot of the cluster graph displayed using Cytoscape-web. Nodes are colored and sized to highlight changes in the user uploaded transcriptome. Larger nodes indicate greater magnitude of change, with green and orange colors indicating up and down regulation, respectively.

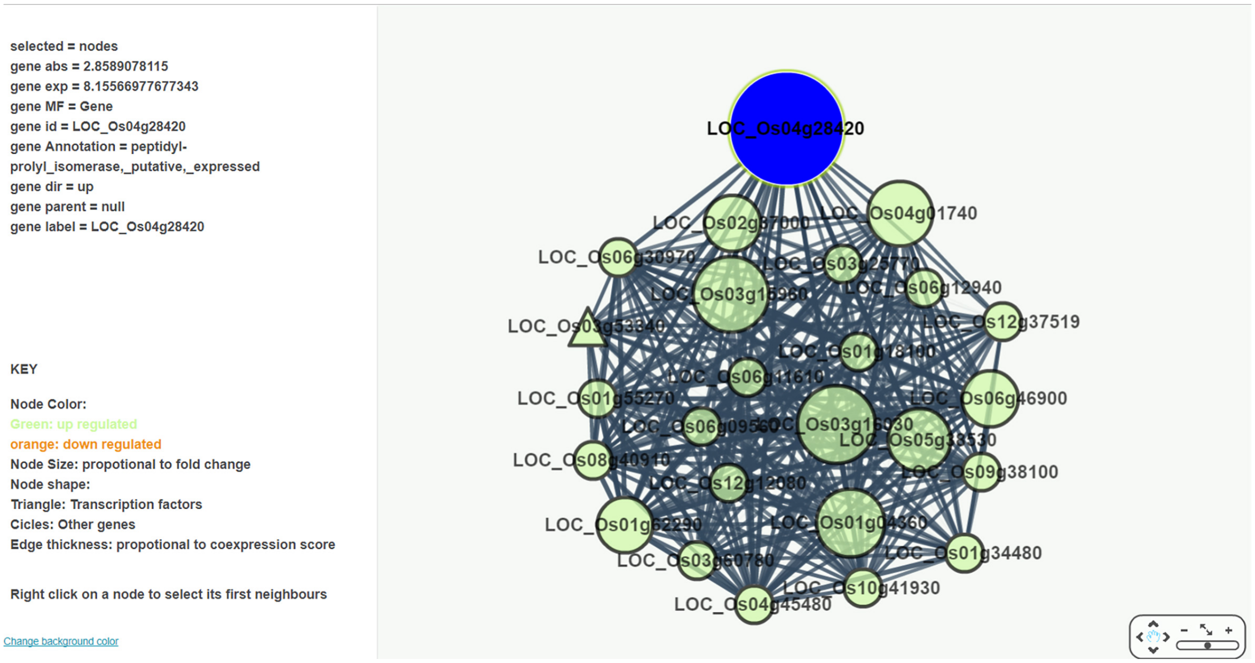

Supplemental Fig. S6

Screenshot of the clusters displayed by searching a biological process.

Clusters enriched in **"response to heat"**

2 clusters found

[click on the cluster ID to see the full list of genes, biological processes, CRE and cytoscape powered graph]

Download this table

	Cluster	Size	GO_ID	No. Genes	Description	-log(qval)
1	Cluster0223	25	GO:0009408	21	response_to_heat	4.1860
2	Cluster0005	972	GO:0009408	21	response_to_heat	2.5205

Supplemental Fig. S7

Screenshot of the BPs enriched in Cluster0005.

	Cluster ID	No. Of Genes
2	Cluster0005	972

Process | **KEGG PATHWAYS** | **Motifs** | **Genes**

Cluster	GO ID	# of Genes	GO Description	Score (-log qvalue)
Cluster0005	GO:0009415	17	response to water stimulus	3.887
Cluster0005	GO:0009628	92	response to abiotic stimulus	3.564
Cluster0005	GO:0006352	47	DNA-dependent transcription initiation	3.111
Cluster0005	GO:0009408	21	response to heat	2.520
Cluster0005	GO:0006367	15	transcription initiation from RNA polymerase II promoter	2.222
Cluster0005	GO:0006396	246	RNA processing	2.216
Cluster0005	GO:0008380	34	RNA splicing	1.821
Cluster0005	GO:0006413	77	translational initiation	1.567
Cluster0005	GO:0006366	15	transcription from RNA polymerase II promoter	1.484
Cluster0005	GO:0006357	17	regulation of transcription from RNA polymerase II promoter	1.386
Cluster0005	GO:0006457	205	protein folding	1.374

Supplemental Fig. S8

Result obtained by querying a single gene.

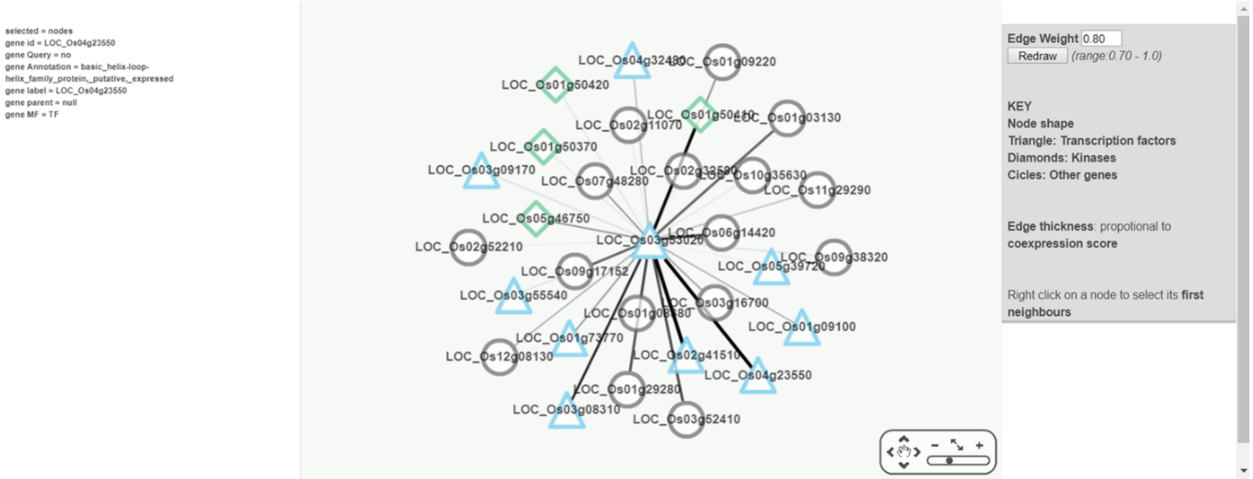

Gene	Annotation	RAP ID	Arabidopsis homolog	Gene details	MF	Seedling	Vegetative	Reproductive
LOC_Os01g03130	expressed protein	Os01g0121500	AT2G01300	see details	Gene	0.454	0.000	0.000
LOC_Os01g08380	transferase family protein, putative, expressed	Os01g0179000	AT3G62160	see details	Gene	0.000	0.000	0.000
LOC_Os01g09100	WRKY10, expressed	Os01g0186000	AT5G26170	see details	TF	0.280	0.976	0.000
LOC_Os01g09220	transposon protein, putative, CACTA, En/Spm sub-class, expressed	Os01g0186900	AT5G12010	see details	Gene	1.519	0.000	0.000
LOC_Os01g29280	expressed protein	Os01g0389200	AT3G02430	see details	Gene	0.000	0.000	0.000
LOC_Os01g50370	STE MEKK ste11 MAP3K.4 - STE kinases include homologs to sterile 7, sterile 11 and sterile 20 from yeast, expressed	Os01g0699100	AT4G26890	see details	Ki	2.320	3.920	0.000
LOC_Os01g50410	STE MEKK ste11 MAP3K.6 - STE kinases include homologs to sterile 7, sterile 11 and sterile 20 from yeast, expressed	Os01g0699500	AT4G26890	see details	Ki	0.994	1.857	0.000
LOC_Os01g50420	STE MEKK ste11 MAP3K.7 - STE kinases include homologs to sterile 7, sterile 11 and sterile 20 from yeast, expressed	Os01g0699600	AT2G32510	see details	Ki	0.000	1.630	0.579
LOC_Os01g73770	dehydration-responsive element-binding protein, putative, expressed	Os01g0968800	AT5G51990	see details	TF	0.000	0.000	0.000
LOC_Os02g11070	3-ketoacyl-CoA synthase, putative, expressed	Os02g0205500	AT2G26640	see details	Gene	0.840	0.000	0.000
